# Editorial: Intrinsically Motivated Open-Ended Learning in Autonomous Robots

**DOI:** 10.3389/fnbot.2019.00115

**Published:** 2020-01-17

**Authors:** Vieri Giuliano Santucci, Pierre-Yves Oudeyer, Andrew Barto, Gianluca Baldassarre

**Affiliations:** ^1^Istituto di Scienze e Tecnologie della Cognizione, Consiglio Nazionale delle Ricerche, Rome, Italy; ^2^Institut National de Recherche en Informatique et en Automatique (INRIA), Bordeaux, France; ^3^College of Information and Computer Sciences, University of Massachusetts, Amherst, MA, United States

**Keywords:** intrinsic motivation, open-ended learning, robotics, developmental robotics, curiosity driven learning

Notwithstanding the important advances in Artificial Intelligence (AI) and robotics, artificial agents still lack the necessary autonomy and versatility to properly interact with realistic environments. This requires agents to face situations that are unknown at design time, to autonomously discover multiple goals/tasks, and to be endowed with learning processes able to solve multiple tasks incrementally and online.

Starting in developmental robotics (Lungarella et al., 2003; Cangelosi and Schlesinger, 2015), and gradually expanding into other fields, intrinsically motivated learning (sometimes called “curiosity-driven learning”) has been studied by many researchers as an approach to autonomous lifelong learning in machines (Oudeyer et al., 2007; Schmidhuber, 2010; Barto, 2013; Mirolli and Baldassarre, 2013). Inspired by the ability of humans and other mammals to discover how to produce “interesting” effects in the environment driven by self-generated motivational signals not related to specific tasks or instructions (White, 1959; Berlyne, 1960; Deci and Ryan, 1985), the research in the field of intrinsically motivated open-ended learning aims to develop agents that autonomously generate motivational signals (Merrick, 2010) to acquire repertoires of diverse skills that are likely to become useful later when specific “extrinsic” tasks need to be performed (e.g., Barto et al., 2004; Baldassarre, 2011; Baranes and Oudeyer, 2013; Kulkarni et al., 2016; Santucci et al., 2016).

This Research Topic aims to present state-of-the-art research on open-ended learning in autonomous robots, with a particular focus on systems driven by intrinsic motivations (but not limited to these systems), and augments the information presented at the *Third International Workshop on Intrinsically Motivated Open-ended Learning – IMOL2017*, held in Rome, Italy, 4–6 October 2017. Although the development of autonomous artificial agents is pursued via different kinds of approaches, such as information theory (Klyubin et al., 2008; Martius et al., 2013), epigenetic robotics (Lones et al., 2016), machine learning (Machado et al., 2017), and evolutionary computation (Lehman and Stanley, 2011), intrinsically motivated open-ended learning is today a mature field producing promising research.

The field nevertheless presents many open challenges, the main ones we mention here. A first open issue of central importance for open-ended learning is how an agent should autonomously generate goals and learn policies for achieving them, so that the policies are useful for solving many new tasks that are unknown when the policies are learned. Another open challenge is to design systems that use intrinsic motivations to support learning compact representations of environment states, and hence of goals; in particular, learning compact representations that are relevant for action. Another challenge involves the continuous/discrete representation of goals. It seems plausible that low-level goals (e.g., related to postures that a robot might assume) are encoded in a continuous space, whereas high-level goals (e.g., touch, push, hit, reach, grasp) are encoded in a discrete fashion so as to be easily composed to achieve more complex goals. If this is the case, what is the relation between these different types of goal representations? A related problem is how to design architectures that can suitably manage generating goals and learning the related skills, and storing them at different levels of granularity. Then there is the open problem of how to best re-use goals and acquired skills to accomplish novel tasks (exploitation), including how to use previous learning in order to efficiently learn new goals and skills (“transfer learning“), and how to form “chains” of interrelated, hierarchical skills (“curriculum learning”), on the basis of intrinsically motivated processes. Learning different tasks in an open-ended fashion tends to cause catastrophic interference, which is another problem that needs to be addressed. Other important challenges are related to the interaction between intrinsic motivations and other forms of “natural learning” such as social interaction: how this interaction might be connected to the development of higher-level cognitive skills, e.g., language.

The contributions collected for this Topic, reviewed below, not only extend the core research in the field but also tackle some of the open questions mentioned above, with a particular focus on: the autonomous acquisition of skills and motor behaviors; the analysis of architectures and learning signals needed to perform sequences of different tasks; the interaction of artificial agents with their environments through different sensors and actuators; the formation and representation of goals; and the interplay between intrinsic motivations and other learning strategies such as imitation learning. Following is a summary of how the contributions in this collection address these challenges.

In Rayyes et al. the authors propose a system enabling a robot to learn to reach to different points in space by exploiting symmetry properties of the actuators to allow exploration to be limited to only a small part of the configuration space. Maestre et al. tackle skill learning at the level of object manipulation, where low-level and high-level features are extracted through task-agnostic interactions with the environment directed toward learning affordances that, in turn, guide the robot to solve assigned tasks. Baldassarre et al. propose a system that uses intrinsic motivations to learn forward models and affordances, here intended as the probabilities of achieving the goals of the affordance-related actions. In particular, this work examines how active-vision, which allows factoring the environment state into pieces of information related to single objects, can support such learning processes and also facilitate solving extrinsic tasks involving multiple objects through one-step planning. Learning progress might also be used as in the contribution of Uchibe to train multiple skills in parallel. In particular, here transfer learning techniques are combined with “mixture of experts” strategies to develop multiple control modules that are then used to solve control tasks with simulated agents. When learning many different tasks, an agent might try to optimize all of them simultaneously. Abdelfattah et al. leverage intrinsic motivations to develop a method that can cope with multi-objective Markov decision processes, and they compare it to other state-of-the-art algorithms. In real environments, complex tasks might need to be learned through exposure to sequences of simpler tasks. This is a crucial issue for autonomous robotics, which is tackled in the work of Duminy et al. using an active learning approach. These authors propose a new algorithm that allows a robot to autonomously discover how to combine pre-defined primitive motor policies to learn increasingly complex combinations of motor policies. In particular, while it is learning, and agent is able to decide which outcome on which to focus and which exploration strategy to apply, leveraging imitation learning, goal babbling, and strategic learning techniques based on intrinsic motivations.

When an agent can autonomously generate different kinds of goals, task-specific reward functions might be complicated to design since they require significant domain knowledge. Intrinsic motivations might provide general, task-agnostic reward functions able to exploit the inherent properties of different goals. Moreover, as shown in Dhakan et al., these reward functions can be used as building blocks to generate sequences of tasks enabling more complex behaviors to be learned. A well-known problem, mentioned above, related to learning multiple tasks is that of catastrophic forgetting. This problem might be even harder to tackle for artificial agents that have to perform life-long learning in complex and unknown environments. Instead of constraining the set of inputs at design time, in Parisi et al., the authors propose a dual-memory self-organizing architecture with two growing recurrent networks that in parallel learn episodic memory and semantic memory, expanding their structures in response to novel sensory experiences.

Vision plays an important role in many aspects related to autonomous learning and exploration. de La Bourdonnaye et al. followed a developmental perspective and built an artificial system that learns to reach for objects in different locations in the environment by leveraging a weakly-supervised stage-wise procedure. Learning to reach is divided into three tasks: learn to fixate objects, learn hand-eye coordination (learn to fixate on the end-effector), and learn to use the previously acquired knowledge to perform reaching to different locations. Visuo-motor coordination is also tackled by Wijesinghe et al., where predictive models are used to guide a humanoid robot in learning to track its hand and other movements in the visual field without the use of any forward kinematics or pre-defined visual feature descriptors. The use of prediction in multi-sensory integration allows a better incorporation of proprioceptive and visual cues and leads to the development of emergent properties similar to those of human hand-eye coordination. Task-agnostic motivations such as information gain are used in the work of Dauce to drive action selection and the exploration of visual inputs: promising results are shown, highlighting how compression strategies might improve both performance in visual recognition and efficiency of the system thanks to reduced computational costs. Autonomous exploration is the focus of Cohen-Lhyver et al. These authors underline the important role of attention, which they claim can be considered as a sort of intrinsic motivation. They implemented this notion in a humanoid robot and showed how two components (congruence and reduction of uncertainty) can be used to explore new environments following audio-visual inputs encoded at a semantic level.

Similar to appealing to information gain, exploration drive by “criticality” can be used to generate autonomous behaviors. Aguilera and Bedia explore this connection through conceptual models that exploit maximum entropy to drive agents toward critical points (e.g., transition points between different kinds of behaviors). Finally, Mahzoon et al. focus on the development of social robots. Within a developmental perspective, these authors address problems related to training real-world robots by presenting two new algorithms that improve a robot's performance in terms of learning efficiency, complexity of the learned behaviors, and predictability of the robot's behavior.

In addition to the aforementioned 14 original research articles, four additional papers have been published within this Research Topic. The first is a methodological article in which Yu et al. present an algorithm that takes into account the constructive interplay between boredom and curiosity, giving rise to effective exploration and forward model learning. The second is a review article in which Khan et al. examine the motivational systems used in computational models to build agents capable of autonomous goal generation and task learning. The authors then investigate how these strategies might be transferred to multi-agent systems and swarms, highlighting the current state-of-the-art and future key challenges. The last two papers are perspective articles. In the first of these, Doncieux et al. argue that a key issue for an agent performing open-ended learning is not only the problem of maximizing the rewards related to the different tasks, but also the problem of building proper representations of the states and the actions describing the tasks themselves. The authors present a conceptual framework to address this crucial issue, underlining the central role of intrinsic motivations. In the second article, Palm and Schwenker analyse the use of reinforcement learning (RL) in the field of developmental robotics, describing its strengths and weaknesses with respect to some specific problems that arise in the field. The authors suggest that multi-objective RL might face some of the problems they listed and that leveraging multiple motivations can improve RL agents' learning performance.

## Author Contributions

All authors listed have made a substantial, direct and intellectual contribution to the work, and approved it for publication.

### Conflict of Interest

The authors declare that the research was conducted in the absence of any commercial or financial relationships that could be construed as a potential conflict of interest.
